# The Case for Reactive Mass Oral Cholera Vaccinations

**DOI:** 10.1371/journal.pntd.0000952

**Published:** 2011-01-25

**Authors:** Rita Reyburn, Jacqueline L. Deen, Rebecca F. Grais, Sujit K. Bhattacharya, Dipika Sur, Anna L. Lopez, Mohamed S. Jiddawi, John D. Clemens, Lorenz von Seidlein

**Affiliations:** 1 International Vaccine Institute (IVI), Seoul, Korea; 2 Epicentre, Paris, France; 3 NICED, Kolkata, India; 4 Ministry of Health and Social Welfare, Zanzibar, Tanzania; 5 Menzies School of Health Research, Casuarina, Australia; Massachusetts General Hospital, United States of America

## Abstract

**Introduction:**

The outbreak of cholera in Zimbabwe intensified interest in the control and prevention of cholera. While there is agreement that safe water, sanitation, and personal hygiene are ideal for the long term control of cholera, there is controversy about the role of newer approaches such as oral cholera vaccines (OCVs). In October 2009 the Strategic Advisory Group of Experts advised the World Health Organization to consider reactive vaccination campaigns in response to large cholera outbreaks. To evaluate the potential benefit of this pivotal change in WHO policy, we used existing data from cholera outbreaks to simulate the number of cholera cases preventable by reactive mass vaccination.

**Methods:**

Datasets of cholera outbreaks from three sites with varying cholera endemicity—Zimbabwe, Kolkata (India), and Zanzibar (Tanzania)—were analysed to estimate the number of cholera cases preventable under differing response times, vaccine coverage, and vaccine doses.

**Findings:**

The large cholera outbreak in Zimbabwe started in mid August 2008 and by July 2009, 98,591 cholera cases had been reported with 4,288 deaths attributed to cholera. If a rapid response had taken place and half of the population had been vaccinated once the first 400 cases had occurred, as many as 34,900 (40%) cholera cases and 1,695 deaths (40%) could have been prevented. In the sites with endemic cholera, Kolkata and Zanzibar, a significant number of cases could have been prevented but the impact would have been less dramatic. A brisk response is required for outbreaks with the majority of cases occurring during the early weeks. Even a delayed response can save a substantial number of cases and deaths in long, drawn-out outbreaks. If circumstances prevent a rapid response there are good reasons to roll out cholera mass vaccination campaigns well into the outbreak. Once a substantial proportion of a population is vaccinated, outbreaks in subsequent years may be reduced if not prevented. A single dose vaccine would be of advantage in short, small outbreaks.

**Conclusions:**

We show that reactive vaccine use can prevent cholera cases and is a rational response to cholera outbreaks in endemic and non-endemic settings. In large and long outbreaks a reactive vaccination with a two-dose vaccine can prevent a substantial proportion of cases. To make mass vaccination campaigns successful, it would be essential to agree when to implement reactive vaccination campaigns and to have a dynamic and determined response team that is familiar with the logistic challenges on standby. Most importantly, the decision makers in donor and recipient countries have to be convinced of the benefit of reactive cholera vaccinations.

## Author Summary

Cholera outbreaks have had catastrophic impact on societies for centuries. Despite more than half a century of advocacy for safe water, sanitation and hygiene, approximately 100,000 cholera cases and 5,000 deaths were reported in Zimbabwe between August 2008 and by July 2009. Safe and effective oral cholera vaccines have been licensed and used by affluent tourists for more than a decade to prevent cholera. We asked whether oral cholera vaccines could be used to protect high risk populations at a time of cholera. We calculated how many cholera cases could have been prevented if mass cholera vaccinations would have been implemented in reaction to past cholera outbreaks. We estimate that determined, well organized mass vaccination campaigns could have prevented 34,900 (40%) cholera cases and 1,695 deaths (40%) in Zimbabwe. In the sites with endemic cholera, Kolkata and Zanzibar, a significant number of cases could have been prevented but the impact would have been less dramatic. The barriers which currently prevent the implementation of mass vaccinations, including but not only the cost to purchase the vaccine, seem insurmountable. A concerted effort of donors and key decision makers will be needed to offer better protection to populations at risk.

## Introduction

In October 2009, the World Health Organization's (WHO) Strategic Advisory Group of Experts (SAGE) on immunization made the pivotal recommendation that oral cholera vaccination should be considered as a reactive strategy in areas with ongoing outbreaks. This is in addition to the continuing recommendation that oral cholera vaccines be used in areas where the disease is endemic and should be considered in areas at risk for outbreaks in conjunction with other prevention and control strategies [1]. Previously, the WHO did not recommend oral cholera vaccination once an outbreak had started due to “the time required to reach protective efficacy and the high cost and heavy logistics associated with its use” [2]. This reluctance has since changed because of the emergence of large and prolonged outbreaks, particularly in sub-Saharan Africa [3]. The large cholera outbreak in Zimbabwe is the latest of these catastrophes [4]. By 2008, 179,323 (94%) of the reported 190,130 cholera cases and 5,074 (99%) of 5,143 cholera deaths reported to the WHO occurred in Africa [5].

There are two oral cholera vaccines (OCVs) available. Dukoral is internationally licensed and prequalified by the WHO for purchase by United Nations agencies and consists of inactivated Vibrio cholerae O1 whole cells combined with the B subunit of the cholera toxin (BS-WC). A large-scale field trial in Bangladesh in the 1980's showed that the BS-WC vaccine is safe and conferred high-grade (∼85%) protective efficacy (PE) during the first 6 months after vaccination, decreasing to ∼60% during the following 18 months and much lower ∼20% in the 3^rd^ year following vaccination [6], [7]. More recently the BS-WC vaccine has been evaluated in Beira, a cholera endemic region of Mozambique, which demonstrated the feasibility and effectiveness of vaccination (PE∼80% during the first year after vaccination) under actual public health conditions in a setting in sub-Saharan Africa [8]. Two more mass vaccination campaigns confirmed the feasibility of this approach in the complex emergency settings of Darfur, Sudan, and in Aceh, Indonesia [9]. A further WHO sponsored evaluation of the BS-WC vaccine is currently under way in Zanzibar. Mass vaccination campaigns with this vaccine in other cholera endemic sites in sub-Saharan Africa are currently under discussion. The BS-WC is administered with a buffer solution in two doses with an interval of at least seven days. Protection is conferred 7 to 10 days after the second dose. The price of this vaccine which is produced in Sweden (approximately USD $18–30/dose on the commercial market) has been a major barrier to wider use. Recently a similar, much less expensive killed OCV has been licensed in Vietnam and subsequently in India [10], [11] and is undergoing WHO prequalification. This second vaccine (WC only), licensed in India as Shanchol, consists of WC without a B subunit and does not require the co-administration of a buffer solution. A double-blind, placebo-controlled trial showed that this vaccine (given in two doses with a minimum inter-dose interval of 14 days) is safe and efficacious, providing nearly 70% protection against clinically significant cholera for at least 2 years after vaccination [12]. Preparations for a trial of a single-dose Shanchol regimen are underway.

The SAGE also recommended that the impact of oral cholera vaccination in halting outbreaks should be documented. In the absence of data from reactive vaccination campaigns we used existing data from cholera outbreaks occurring in endemic and non-endemic settings in Asia and Africa to compare actual cholera outbreaks with simulated outbreaks during which a mass vaccination takes place. To add realism to our simulation we have varied the response times, and made estimates for single- and two-dose vaccines.

## Materials and Methods

### Sites

Three sites in three countries Zimbabwe, Kolkata (India), and Zanzibar (Tanzania) were selected based on a) availability of reliable data and b) absence of interventions such as vaccinations. The sites vary in cholera endemicity (Table 1). In Kolkata cholera is endemic and seasonal cholera outbreaks can be predicted. Cholera is also endemic in Zanzibar, however the location of cholera clusters tends to shift between years and is not easily predictable. In Zimbabwe cholera is not yet endemic. Large cholera outbreaks occurred in 1999 and 2002 [5]. Between 2002 and 2008 no cholera outbreaks were reported. In Zanzibar and Zimbabwe the large majority of cholera cases were clinically diagnosed. In Kolkata all cholera cases were laboratory-confirmed. A detailed description of the Kolkata site in has been published [13].

**Table 1:** The study sites.

	Zimbabwe	Zanzibar, Tanzania	Kolkata, India
Area under consideration	Whole country	Unguja and Pemba	Ward 29, 30
Cholera endemicity	epidemic	endemic	endemic
Population	13,4 million	1,182,804	57,099
Annualized cholera incidence	-	0.5 per 1000	1.6 per 1000
Attack rate	7.39/1000	-	-
Laboratory confirmation	First cases only	First cases only	all

Zimbabwe is a republic located in the southern part of Africa. The population of the country is approximately 13,4 million. The cholera outbreak in 2008/9 is one of the largest outbreaks ever recorded. The outbreak started in August 2008, lasted for 49 weeks and affected all 10 of the country's provinces. The data used in our study comes from the daily cholera updates posted by United Nations Office for the Coordination of Humanitarian Affairs (OCHA), Zimbabwe [14]. The large majority of reported cases were based on clinical diagnosis. The daily updates were entered in an excel spreadsheet and analysed. Since daily updates were only posted from November 2008 onwards we extrapolated the epidemic curve backwards between August, the date the first cases were reported, and November 2008 when daily reporting started. The country-wide attack rate during 49 weeks of the 2008/9 outbreak was 7.4 per 1000 population.

The site in India consists of legally registered urban slum areas (bustees) within the administrative wards 29 and 30 in the city of Kolkata [13]. The area has a high population density and residents do not have sufficient water supply or sanitary facilities. A baseline census of the study population was done in early 2003 and enumerated 57,099 individuals. Study site residents of all ages were under surveillance for diarrhoea treated at any of the five project health clinics set up in the field and the city's infectious diseases and children's hospitals. Rectal swabs in Cary-Blair media were brought on the same day from the project health outposts to the study laboratory at the National Institute of Cholera and Enteric Diseases. Vibrio cholerae were isolated and identified using standard methods. The annualized cholera incidence during the surveillance period from May 2003 to April 2005 was 1.6 per 1000 population.

In Zanzibar, the site consists of two islands Unguja and Pemba, 40km and 60km off mainland Tanzania, with a total population of 1,182,804 in 2008, calculated from 2002 national census data. In Zanzibar the first cholera cases were detected in 1978. For this study the seven outbreaks occurring in Unguja and Pemba during the decade 1997 to 2007 were reviewed. In Unguja, cholera patients tend to reside in densely populated urban areas with water supply from communal and private taps. In contrast, in Pemba outbreaks were mainly reported in rural areas, with shallow wells as the primary water source. Routine surveillance reports completed by the national surveillance system were reviewed. The majority of cases are clinically diagnosed with the initial cases laboratory confirmed using standard methods, at Mnazi Mmoja Hospital in Unguja, and the Public Health Laboratory Ivo de Canieri in Pemba. Between 1997 and 2007, 5640 cholera cases were reported in Zanzibar, assuming a population of 1,037,183 during this period, the annualised incidence was 0.5 per 1000 population over the decade.

### Definitions and assumptions

Outbreaks are defined as temporally- and geographically-clustered cholera cases, separated by the absence of cases for a minimum of 6 months. We assume that the vaccine confers 85% protection during the first 6 months following vaccination decreasing to 60% up to the end of year 2 and 20% protection in the 3^rd^ year following vaccination. We define vaccination as the ingestion of the OCV and immunisation as the protective biologic response following vaccination (Table 2). Administration of two vaccine doses either at 7, 14 or 28–42 day intervals results in similar immune responses after the second dose [15]. Protection against cholera can be expected to start one week after the primary immunization series.

**Table 2:** Estimated response times to mount a mass oral cholera vaccination campaign, using a two-dose vaccine.

	Response Period	Range	Rapid	Delayed	Maximum
1	An outbreak starts	1st reported case	1st reported case	1st reported case	1st reported case
2	An outbreak is recognized	24h–6 wks	24h	3 wks	6 wks
3	Agreement to send vaccine	24h–6 wks	24h	3 wks	6 wks
4	Vaccine shipment arrives	2 wks–6 wks	2 wks	4 wks	6 wks
5	Vaccinations start - first dose	1 wks–4 wks	1 wk	2 wks	4 wks
6	First dose is completed	2 wks–4 wks	2 wks	3 wks	4 wks
7	Minimal delay between first and second dose	1 wk	1 wk	1 wk	1 wk
8	Second dose starts	1 wk	1 wk	1 wk	1 wk
9	Vaccinations completed	2 wks–4 wks	2 wks	3 wks	4 wks
10	Community immunized	1 wk after last dose	1 wk after last dose	1 wk after last dose	1 wk after last dose
	Total response time		10 wks	21 wks	33 wks

We assume the availability of a cholera vaccine through a rotating stockpile to prevent the expiration of doses. The overall response time is divided into the following components, the outbreak is recognized, an agreement to send vaccine is reached, the vaccine shipment arrives, vaccinations start, the administration of the first dose is completed, delay between first and second dose, second dose starts, vaccinations are completed, and finally the participants are immunized (Table 2 and 3). There is currently no agreement on a threshold to trigger cholera vaccination campaigns. We assume that in the endemic settings such as Kolkata or Zanzibar, the time from the report of the initial cholera cases to the recognition that an outbreak is occurring could take between 24 hours to 6 weeks, the number of cases reported before an outbreak was recognised under these assumptions is displayed in Table 4. In contrast the outbreak in Zimbabwe was in a non-endemic setting and orders of magnitude bigger. We arbitrarily set the threshold which should have triggered a vaccination campaign in Zimbabwe at 400 cases. The period required for the stockpile administrators to come to an agreement to implement a mass oral cholera vaccination and ship the vaccine could also take between 24 hours and 6 weeks. The vaccine shipment via air courier could take between 2 and 6 weeks depending on the urgency as well as potential delays clearing customs. Setting up the posts and starting vaccination will take 1 to 4 weeks and completion of the first dose will take 2 to 4 weeks. An interval of at least 7 days is required between the two doses. Starting the administration of the second dose will require up to 7 days, assuming staff and materials are on stand-by following the first dose. Completion of the second dose will take 2 to 4 weeks. The time required for vaccinated individuals to mount an immune response is 7 days. In total, the minimum time required to immunize the community is about 10 weeks, a delayed response would be 21 weeks and in the worst case scenario, a maximum response time could take as long as 33 weeks (Table 2).

**Table 3:** Estimated response times to mount a mass oral cholera vaccination campaign, using a single-dose vaccine.

	Response Period	Range	Rapid	Delayed	Maximum
1	An outbreak starts	1st reported case	1st reported case	1st reported case	1st reported case
2	An outbreak is recognized	24h–6 wks	24h	3 wks	6 wks
3	Agreement to send vaccine	24h–6 wks	24h	3 wks	6 wks
4	Vaccine shipment arrives	2 wks–6 wks	2 wks	4 wks	6 wks
5	Vaccinations start	1 wks–4 wks	1 wk	2 wks	4 wks
6	Vaccinations completed	2 wks–4 wks	2 wks	3 wks	4 wks
7	Community immunized	1 wk after last dose	1 wk after last dose	1 wk after last dose	1 wk after last dose
	Total response time		6 weeks	16 weeks	27 weeks

**Table 4:** Cases which had been reported before the outbreak was recognised, initiating potential reactive vaccination efforts.

Site	Year	No. of cholera cases reported before the outbreak was recognised		
		Rapid response time (24hrs after the first case)	Delayed (3 weeks after the first case)	Maximum (6 weeks after the first case)
Kolkata	2003	3	3	6
	2004	1	51	84
	2005	1	4	13
Unguja	1997–98	14	142	164
	2002–03	14	62	119
	2004–05	43	178	322
	2006–07	2	73	154
Pemba	2002–03	6	28	81
	2003–04	15	64	171
	2006–07	21	124	167

In Zimbabwe 24 hrs, 3 weeks and 6 weeks after the first 400 cases had been reported (and the outbreak recognised) the total number of cumulative cases were 501, 1,401 and 3,501 respectively (i.e. including the first 400).

The use of a hypothetical single-dose vaccine with similar protective efficacy and duration of protection as the licensed two-dose vaccine will reduce the response period, as the minimal delay between first and second dose and the time to complete the second dose will no longer apply. The use of single-dose vaccine would reduce the rapid response time in our simulations from 10 weeks to 6 weeks (a 40% reduction), the delayed response time from 21 to 16 weeks (a 24% reduction) and finally a maximum response time from 33 to 27 weeks, (an 18% reduction, Table 3).

### Ethics

In compliance with Good Clinical Practice guidelines all information that could reveal the identity of study participants was removed prior to analysis. None of the data available to the investigators of the submitted study contained information which could potentially reveal the identity of the participants. The data from Zimbabwe was public domain data so ethics approval was is not necessary. The data from Kolkata was collected in preparation of large cholera vaccine trials. It was considered sufficient and appropriate by the local investigators to obtain verbal, informed consent since the participation in the study consisted of taking a medical history, physical examination, testing of stool specimens. All procedures included in the study participation are part of good routine management of diarrhea patients. No experimental procedures of any kind were conducted on study participants. Besides the local (ethics committee of the National Institute of Cholera and Enteric Diseases) and the national ethics review committee (the Health Ministry Screening Committee of India), the study was approved by the WHO Secretariat Committee on Research Involving Human Subjects and the International Vaccine Institute Institutional Review Board. The data from Zanzibar was collected in preparation for a large cholera vaccine effectiveness study. Data was summarised routine surveillance data without any patient identifiers and so individual patient consent was unobtainable and unnecessary. Permission to use the data was granted by the Ministry of Health and Social Welfare, Zanzibar, who provided the data for the study.

### Data analysis and modelling

We created graphs of each outbreak showing number of cholera cases by week. The number of prevented cases (PC) was calculated as the product of the number of reported cases (RC), protective efficacy (PE), and percent of the population participating in mass vaccination campaigns (Vaccine coverage, VC) or PC = RC×PE×VC. PE was set at 85% during the first six months after vaccination, 60% after 6 months and 20% in the third year based on previously published data [6], [7]. We assumed that between 50% and 75% of the target population will participate in mass vaccination campaigns. VC was therefore set at 50% and repeated at 75%. The number of prevented cases was subtracted from the reported number of cases to model the outbreak curves after mass vaccinations. The number of prevented cases was calculated for the varying response times shown in Table 2 for a two-dose vaccine and in Table 3 for a single-dose vaccine.

## Results

### The effect of mass vaccination using the two-dose OCV

**Figure 1:**
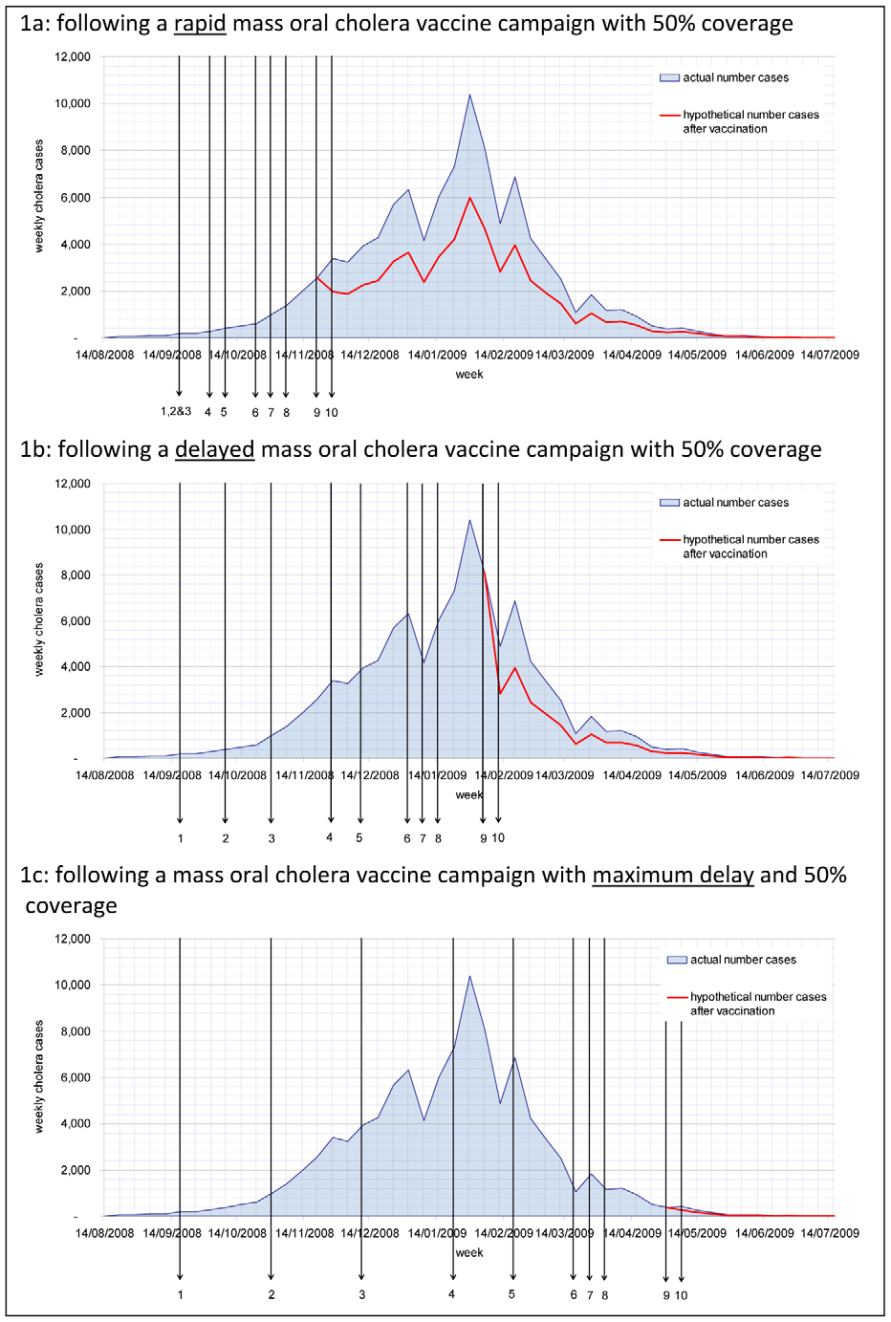
Estimated reduction in cholera cases during the Zimbabwe 2008–09 outbreak.* 1a: Following a rapid mass oral cholera vaccine campaign with 50% coverage. 1b: Following a delayed mass oral cholera vaccine campaign with 50% coverage. 1c: Following a mass oral cholera vaccine campaign with maximum delay and 50% coverage. *See Table 2 for the time point symbolized by each arrow. Figures show the epicurve of the outbreak and the hypothetical number of cases prevented at response time.

In Zimbabwe the first cholera cases were reported in August 2008 [5]. The outbreak reached its peak in the last week of January 2009 and had subsided by the beginning of July 2009. By July 2009, 98,591 cholera cases had been reported with 4,288 deaths attributed to cholera. The overall case fatality rate (CFR) was 4% with no significant decrease in CFR throughout the outbreak. If a stockpile of cholera vaccines would have been available mass vaccinations could have been implemented once a critical number of cholera cases had been diagnosed. The outbreak affected the whole of Zimbabwe hence a nationwide vaccination campaign would have been required. We calculated the reduction in cases that a two-dose vaccine would have achieved under different conditions (Figs 1a to c and Table 5). Had a rapid response taken place after the initial 400 cases had been reported, we estimate that as many as 34,900 of 98,591 (40%) cholera cases and 1,695 of 4,288 (40%) deaths could have been prevented by a mass OCV campaign with 50% coverage (Fig 1a and Table 5). Delayed and maximum time responses would have resulted in fewer cases prevented (Figs 1b and c and Table 5).

**Table 5:** Cases that would have been prevented by mass oral vaccination oral cholera vaccine in three endemic sites.

Site	Year	Population	Outbreak duration	Total cholera cases	Attack rate/ 1000	No. (%) cholera cases prevented at variable response times with 50% vaccine coverage			No. (%) cholera cases prevented at variable response times with 75% vaccine coverage		
						Rapid	Delayed	Maximum	Rapid	Delayed	Maximum
Zimbabwe	2008–09	13,349,000	54 wks	98,591	7.39	34,900 (40)	12,789 (13)	474 (0)	59,100 (60)	19,183 (19)	711 (1)
Kolkata	2003		30 wks	53	0.91	19 (36)	7 (13)	0 (0)	29 (54)	10 (19)	0 (0)
	2004	58,063	36 wks	136	2.34	18 (13)	14 (10)	3 (3)	27 (20)	21 (15)	5 (4)
	2005		41 wks	33	0.59	8 (23)	5 (14)	1 (3)	11 (35)	7 (21)	1 (4)
Unguja	1997–98	534,512	35 wks	452	0.85	108 (24)	73 (16)	6 (1)	162 (36)	110 (24)	8 (2)
	2002–03	643,905	88wks	687	1.07	164 (24)	163 (24)	162 (24)	246 (36)	243 (35)	243 (35)
	2004–05	692,591	36wks	286	0.41	57 (20)	45 (16)	7 (3)	86 (30)	67 (23)	11 (4)
	2006–07	745,262	63 wks	1974	2.65	558 (28)	610 (31)	546 (28)	837 (42)	915 (46)	819 (41)
Pemba	2002–03	368,910	14wks	119	0.32	5 (4)	0 (0)	0 (0)	8 (6)	0 (0)	0 (0)
	2003–04	377,206	55 wks	862	2.29	253 (29)	261 (30)	124 (14)	379 (44)	391 (45)	186 (22)
	2006–07	403,229	75 wks	1260	3.12	304 (24)	217 (17)	212 (17)	457 (36)	326 (26)	317 (25)

Mass vaccination is presumed to be using the currently licensed two-dose oral cholera vaccine at varying response times and vaccine coverage.

**Figure 2:**
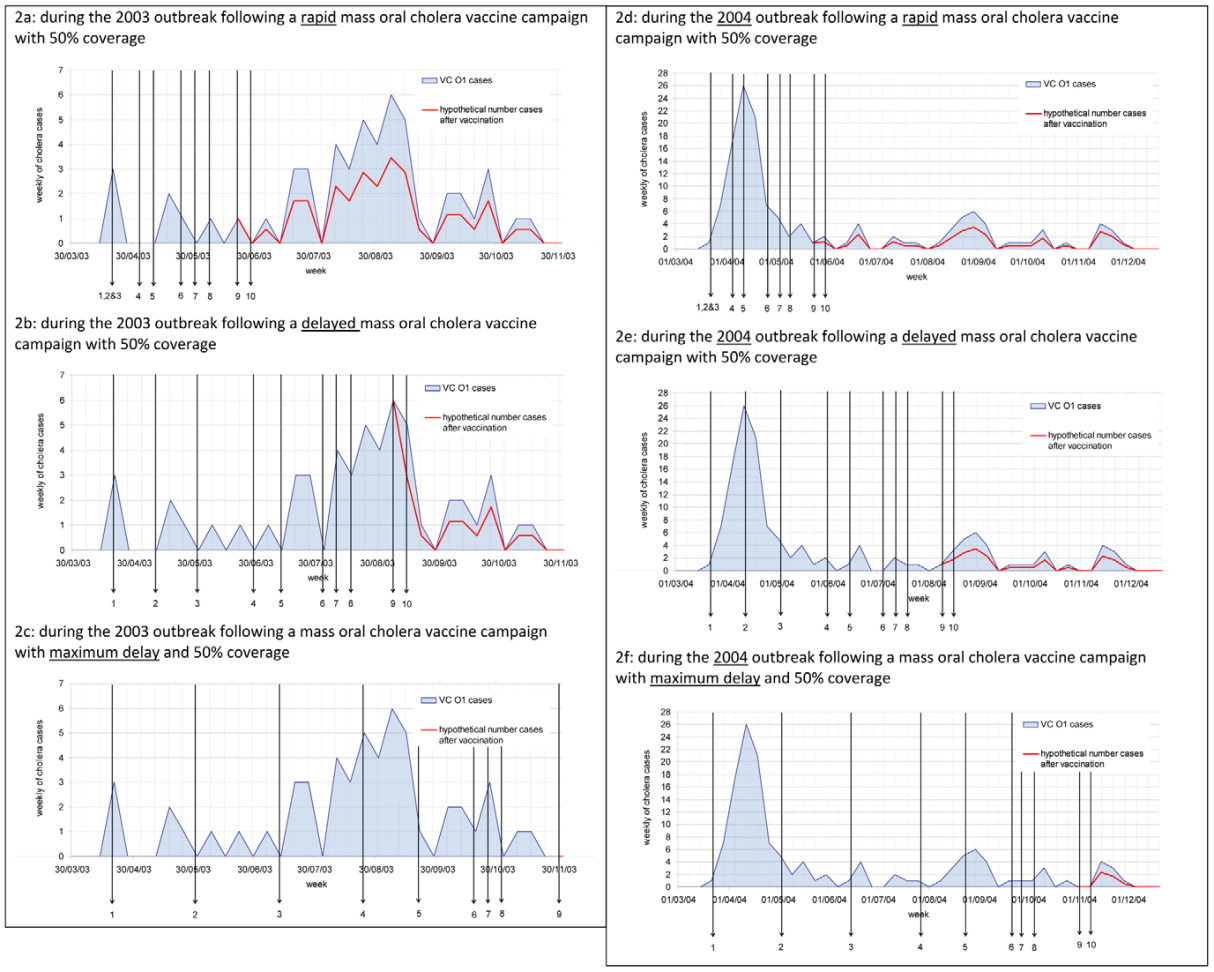
Estimated reduction in cholera cases in Kolkata.* 2b: During the 2003 outbreak following a delayed mass oral cholera vaccine campaign with 50% coverage. 2a: During the 2003 outbreak following a rapid mass oral cholera vaccine campaign with 50% coverage. 2c: During the 2003 outbreak following a mass oral cholera vaccine campaign with maximum delay and 50% coverage. 2d: During the 2004 outbreak following a rapid mass oral cholera vaccine campaign with 50% coverage. 2e: During the 2004 outbreak following a delayed mass oral cholera vaccine campaign with 50% coverage. 2f: During the 2004 outbreak following a mass oral cholera vaccine campaign with maximum delay and 50% coverage. *See Table 2 for the time point symbolized by each arrow. Figures show the epicurve of the outbreak and the hypothetical number of cases prevented at response time.

In Kolkata three outbreaks in 2003, 2004, and 2005 were reviewed. During the 2003 outbreak, 53 cases were detected. The number of cholera cases that could have been prevented by reactive mass vaccination with a coverage of 50%, was between 19 (36%) with a rapid response time to as low as 7 (13%) with a delayed response time (Fig 2a and b and Table 5). With a higher participation rate of 75%, which is realistic in this setting, the number of cholera cases avoided increased to 29 (54%) with a rapid response time (Table 5). There would be no reduction of cases associated with a maximum response time of 33 weeks, as the 2003 outbreak lasted only 30 weeks (Figure 1c). During the 2004 outbreak, 136 cases were detected. The number of cholera cases that could have been prevented by reactive mass vaccination with a coverage of 50%, was between 18 (13%) with a rapid response, 14 (10%) with a delayed response and 3 (3%) with the maximum response time (Fig 2d and e and Table 4). The number of cholera cases which could have been prevented in the 2005 season is shown in Table 5, the number of cases reported before an outbreak is recognised is shown in Table 4.

**Figure 3:**
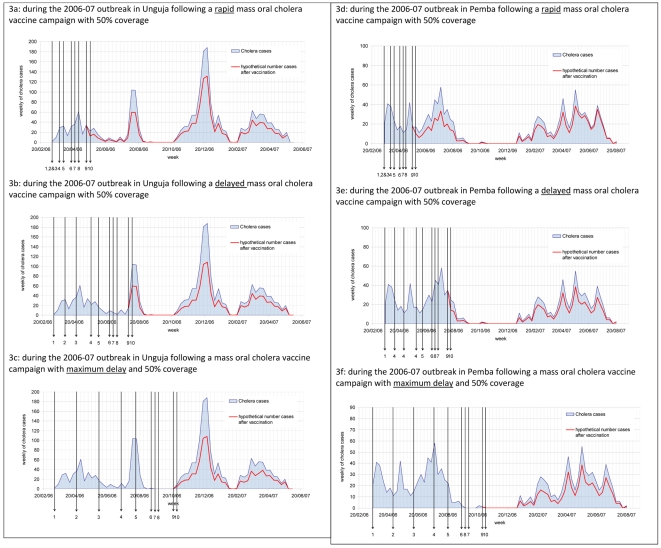
Estimated reduction in cholera cases in Zanzibar.* 3a: During the 2006–07 outbreak in Unguja following a rapid mass oral cholera vaccine campaign with 50% coverage. 3b: During the 2006–07 outbreak in Unguja following a delayed mass oral cholera vaccine campaign with 50% coverage. 3c: During the 2006–07 outbreak in Unguja following a mass oral cholera vaccine campaign with maximum delay and 50% coverage. 3d: During the 2006–07 outbreak in Pemba following a rapid mass oral cholera vaccine campaign with 50% coverage. 3e: During the 2006–07 outbreak in Pemba following a delayed mass oral cholera vaccine campaign with 50% coverage. 3f: During the 2006–07 outbreak in Pemba following a mass oral cholera vaccine campaign with maximum delay and 50% coverage. *See Table 2 for the time point symbolized by each arrow. Figures show the epicurve of the outbreak and the hypothetical number of cases prevented at response time.

In Unguja, Zanzibar four outbreaks were reviewed. From the 52^nd^ week of 1997 to the 34^th^ week of 1998 a total of 452 cases were detected (0.85 cholera cases/1000 population). A reactive island-wide mass vaccination campaign with 50% vaccine coverage would have prevented 108 (24%) cholera cases with a rapid response and 6 (1%) cases with the maximum response time. With a 75% vaccine coverage, the number of cholera cases prevented would have been 162 (36%) with a rapid response time to 8 (2%), with the maximum response time. To initiate a rapid response an outbreak would be recognised after 24 hours, when 14 cases had been reported, in a maximum response time an outbreak would have been recognised after 6 weeks after a total of 164 cases had been reported (Table 4). The number of cholera cases which could have been prevented in three subsequent outbreaks is shown in Table 5. In Pemba three outbreaks were reviewed. A total of 119 cholera cases were detected (0.32 cholera cases/1000 population) from the 50^th^ week of 2002 to the 12^th^ week of 2003. A reactive island-wide mass vaccination campaign with 50% vaccine coverage would have prevented 5 (4%) cholera cases with a rapid response time. Delayed and maximum response times did not prevent any cholera cases. The following year the number of preventable cases increased to 253 (29%) and 124 (14%) respectively for rapid and maximum response times. The number of cases that could be avoided during the following years and under different assumptions is shown in Table 5, and the number of cases reported before recognising an outbreak shown in Table 4. Figure 3a to 3f shows the number of preventable cases in Unguja and Pemba during the 2006–2007 outbreak had reactive vaccination been employed under different assumptions.

### Protection in subsequent years

While the protective efficacy is waning in the years following immunisation there remains ∼60% PE during the following 18 months and ∼20% PE in the 3^rd^ year following vaccination. More cases are prevented in years following maximum and delayed responses vaccinations as there is no delay in protection. In endemic settings a reactive vaccination campaign using a the two-dose OCV in the first outbreak with 50% coverage in Zanzibar, would prevent 137–168 cases in Unguja and 184–189 cases in Pemba during the three years following a rapid response and maximum response vaccination campaigns, respectively (Table 6). In Kolkata 43–55 cases would be prevented during the three years following a rapid response and maximum response vaccinations, respectively.

**Table 6:** The number of cases that would have been prevented during the initial outbreak and in the years following vaccination.

Site	Year	Population	Outbreak duration	Total cholera cases	Attack rate/ 1000	The total numbers (%) of cholera cases prevented		
						Rapid	Delayed	Maximum
Kolkata	2003–05	58,063	142 wks	223	3.84	62 (28)	54 (24)	55 (25)
Unguja	2004–07	643,905	136 wks	2260	3.51	301 (13)	316 (14)	330 (15)
Pemba	2002–04	368,910	89 wks	750	2.03	189 (25)	189 (25)	189 (25)

The total number of cases which would have been prevented during the first outbreak and in the years following mass vaccination using the currently licensed two-dose oral cholera vaccine with 50% vaccine coverage, and 85% PE in first 6 months, 60% PE from 6 to 18 months, and 20% for the following 3 years, at variable response times.

### The effect of mass vaccination using a single-dose vaccine

If a hypothetical single-dose vaccine with similar characteristics as the two-dose vaccine would have been available for a rapid mass vaccination in Zimbabwe, 41,059 cases could have been prevented (42%) and 1,748 deaths (41%; Table 7). In three other sites a single-dose vaccine in a rapid response vaccination could have prevented a total of 1,768 cases (29%), reducing outbreak size by 3% compared to a two-dose vaccine.

**Table 7:** Cases that would have been prevented by mass vaccination using a hypothetical single-dose oral cholera vaccine.

Site	Year	Population	Outbreak duration	Total cholera cases	Attack rate/ 1000	No. (%) cholera cases prevented at variable response times with 50% vaccine coverage			No. (%) cholera cases prevented at variable response times with 75% vaccine coverage		
						Rapid	Delayed	Maximum	Rapid	Delayed	Maximum
Zimbabwe	2008–09	13,349,000	54 wks	98,591	7.39	41,059 (42)	28,075 (28)	3,038 (3)	61,589 (62)	42,112 (43)	4,557 (5)
Kolkata	2003		30 wks	53	0.91	20 (37)	16 (30)	2 (4)	30 (56)	24 (47)	3 (6)
	2004	58,063	36 wks	136	2.34	23 (17)	16 (12)	16 (4)	35 (25)	24 (18)	9 (7)
	2005		41 wks	33	0.59	9 (27)	6 (19)	3 (10)	13 (40)	10 (29)	5 (15)
Unguja	1997–98	534,512	35 wks	452	0.85	124 (27)	84 (19)	53 (12)	185 (41)	126 (28)	80 (18)
	2002–03	643,905	88wks	687	1.07	195 (28)	142 (21)	161 (23)	292 (42)	213 (31)	242 (35)
	2004–05	692,591	36wks	286	0.41	88 (31)	45 (16)	28 (10)	132 (42)	68 (24)	42 (15)
	2006–07	745,262	63 wks	1974	2.65	621 (31)	546 (28)	515 (26)	931 (47)	820 (42)	773 (39)
Pemba	2002–03	368,910	14wks	119	0.32	22 (18)	0 (0)	0 (0)	33 (27)	0 (0)	0 (0)
	2003–04	377,206	55 wks	862	2.29	267 (31)	266 (31)	216 (25)	400 (46)	399 (46)	324 (38)
	2006–07	403,229	75 wks	1260	3.12	315 (25)	285 (23)	202 (16)	473 (38)	427 (34)	303 (24)

Varying response times and vaccine coverage are shown for the three sites.

## Discussion

We found that the number of cholera cases prevented by reactive mass vaccination campaigns depends on the size and shape of the outbreak curve. Reactive mass vaccinations can be expected to be most effective in large, long-lasting outbreaks which are most likely to occur in populations with no past exposure, i.e. where cholera appears de novo or returns after a long period of absence. In the presence of a sufficiently large, susceptible population an outbreak can continue for months. The outbreak in Zimbabwe petered out approximately 11 months after the first cases were reported. It has been estimated previously that cholera outbreaks in a refugee camp last 20 weeks hence reactive vaccination was unlikely to be cost–effective [16]. The large and long cholera outbreak in Zimbabwe demonstrated a different dynamic and lasted more than twice as long as average outbreaks in refugee camps. A reactive cholera vaccination campaign would have prevented significant numbers of cholera cases and deaths. In contrast during shorter outbreaks in the endemic settings of Kolkata and Zanzibar, where the majority of cases occur early on, only an immediate, brisk response will prevent a substantial number of cases. A second important finding was the minimal advantage of a single-dose in reducing the number cholera cases compared to a two-dose vaccine regimen. The advantage of single dose vaccines was most pronounced in small short outbreaks and less important in larger and longer outbreaks. A single dose vaccine would increase coverage as there would be no drop out between the first and the second dose.

Public health experts have considered establishing a cholera vaccine stockpile similar to the existing yellow fever and the meningococcal vaccine stockpiles [17], [18]. A concerted effort to distribute a hypothetical cholera vaccine stockpile could have potentially prevented more than a third of the cholera cases and deaths in Zimbabwe 2008–9. However there is currently no cholera vaccine stockpile in existence which the international aid community could have used for this purpose. Secondly there is a consensus opinion that the political situation in Zimbabwe at the time of the outbreak would have prevented mass vaccination campaigns. Thirdly not all provinces were initially affected by the cholera outbreak. Early strategically targeted mass vaccination campaigns potentially could have prevented the spread of cholera which ultimately affected all provinces. Important lessons could and should be learned from this disaster for future cholera outbreaks.

A major challenge for the administration of a cholera vaccine stockpile will be a consensus on the number of cholera cases which represent the threshold to trigger mass vaccination campaigns. The threshold should discriminate between sporadic cholera cases and an outbreak. This threshold may have to be calibrated for the cholera endemicity and the dynamics of outbreaks. The number of cholera cases may have a different significance in a cholera endemic area compared to an area where cholera hasn't been detected for several years. A steady increase in the daily number of reported cases is more worrying than stable or a declining numbers of daily cholera cases. In the absence of a consensus of a threshold to start cholera mass vaccinations we have assumed for the purpose of this paper that it may take between 24 hours and 6 weeks until a threshold is reached which triggers a mass vaccination.

We have underestimated the benefit of vaccinations as we could not include the added benefit of herd immunity which is likely to further reduce the number of cholera cases and deaths. Widespread administration of cholera vaccines protects the vaccinated individual as well as the unvaccinated community members with indirect protection proportional to vaccine coverage [19]. Longini and co-workers have used modelled data to show that cholera transmission could be controlled in endemic areas with 50% coverage with OCVs [20]. However the available evidence for herd immunity is based on data collected in a randomised controlled trial conducted in Bangladesh in the 1980s where cholera was endemic. We have currently no data on the added protection conferred by herd immunity following reactive vaccinations in cholera outbreaks. It seems likely that herd immunity will have an additive effect in reactive vaccinations. There is hope that a vaccine coverage of 50% or more may reduce the basic reproductive number below equity and abort cholera outbreaks altogether. However without empiric evidence it is highly speculative trying to quantify the added protection conferred by herd immunity. Estimating such added protection conferred through herd immunity would be highly informative for future mass vaccination campaigns.

Another limitation of this study was the selection of culture confirmed cases from Kolkata. It seems likely that cholera cases were not captured because they did not present to the treatment centre or had false negative microbiology results. The true number of cases is most likely higher. For our calculations, we used a minimum inter-dose interval of 7 days which is recommended for the BS-WC vaccine, whereas efficacy data is available only for an inter-dose interval of 14 days for the WC-only vaccine [12]. Due to the expected dropout between the first and second dose it may be necessary to vaccinate 10 to 20% more people to achieve a coverage of 50% or 75% with two complete doses. However the 14 day interval may not be essential as immunogenicity data suggests protection starting even after a single dose [21], [22]. Finally our database may not be representative of other cholera outbreaks. However in the absence of surveillance, other available epidemic curves are likely to be incomplete, especially missing the cases at the start of the outbreak. Furthermore the Kolkata data comes from a restricted population which may have underestimated outbreak duration compared to Zimbabwe and Zanzibar, where data were collected from the whole population. Clearly our models do not provide precise predictions of the number of cases prevented but they serve to highlight questions and point to areas where further research is needed.

As it is currently impossible to predict the size and shape of an outbreak curve, our data suggest that time is of the essence, a brisk response will provide most benefit. If circumstances prevent a rapid response there remain good reasons to roll out cholera mass vaccination campaigns in response to an outbreak report. Even if the vaccination does not impact greatly the current outbreak, once a substantial proportion of a population is vaccinated, outbreaks in subsequent years may be reduced if not prevented. This benefit should be taken into consideration when deciding on reactive cholera vaccinations. Furthermore containment of the disease in one area may prevent the spread to other susceptible populations. Because outbreaks are heterogeneous a large number of outbreaks would have to be randomized to assess the impact of reactive vaccinations. Hence a meaningful assessment of the impact of reactive mass vaccinations may not be feasible. Modelling the cost effectiveness of reactive mass vaccinations may well provide further evidence of the benefit of this intervention.

Our findings support the SAGE recommendations to include reactive mass vaccination campaigns in the interventions to manage cholera outbreaks. An explosive cholera outbreak in Haiti at the end of 2010, where a population of 10.1 million people has no prior immunity has added poignancy to our report [23]. There is an urgent need for financial mechanisms to establish and maintain a stockpile of cholera vaccines as well as a determined and dynamic team to administer such a stockpile. Perhaps most importantly decision makers in affected countries have to become aware of the benefit of reactive vaccination campaigns and actively promote their use.
